# Inorganic carbon addition stimulates snow algae primary productivity

**DOI:** 10.1038/s41396-018-0048-6

**Published:** 2018-01-29

**Authors:** Trinity L. Hamilton, Jeff R. Havig

**Affiliations:** 1 Department of Plant and Microbial Biology, University of Minnesota, St. Paul, MN 55108 USA; 2 Department of Earth Sciences, University of Minnesota, Minneapolis, MN 55455 USA

**Keywords:** Water microbiology, Biogeochemistry, Climate-change impacts, Biogeochemistry

## Abstract

Earth has experienced glacial/interglacial oscillations accompanied by changes in atmospheric CO_2_ throughout much of its history. Today over 15 million square kilometers of Earth’s land surface is covered in ice including glaciers, ice caps, and ice sheets. Glaciers are teeming with life and supraglacial snow and ice surfaces are often darkened by the presence of photoautotrophic snow algae, resulting in accelerated melt due to lowered albedo. Few studies report the productivity of snow algal communities and the parameters which constrain their growth on supraglacial surfaces—key factors for quantifying biologically induced albedo effects (bio-albedo). We demonstrate that snow algae primary productivity is stimulated by the addition of inorganic carbon. Our results indicate a positive feedback between increasing CO_2_ and snow algal primary productivity, underscoring the need for robust climate models of past and present glacial/interglacial oscillations to include feedbacks between supraglacial primary productivity, albedo, and atmospheric CO_2_.

Earth has experienced intervals of glacial and interglacial periods in its history including Snowball Earth events [1, 2]. Today, glaciers and ice sheets are integral to the Earth’s climate and hydrological system—they influence regional and global climate, are sensitive to climate change, and are the largest freshwater reservoir on Earth [3, 4]. Geologic and geochemical evidence suggest that glacial/interglacial oscillations are coincident with lower atmospheric CO_2_ [5] and are exacerbated by lower solar luminosity [6]. For instance, models indicate overcoming high planetary albedo during Snowball Earth events required greenhouse warming caused by the accumulation of high levels of CO_2_ from volcanic outgassing accompanied by decreases in silicate weathering [7, 8]. Due to human activity, atmospheric CO_2_ is now above 400 ppm [9] and from 1999 to 2010, CO_2_ was emitted at a rate 100 times as fast as during the last glacial termination [10]. Coincident with increasing CO_2_, average global temperatures have increased (~1 °C over the past century) leading to glacial retreat and receding snowpack.

Glaciers and ice sheets are a host to diverse ecosystems including supraglacial communities that contribute to local and global biogeochemical cycles [11]. Snow algae (eukaryotic photoautotrophs) are key primary producers on supraglacial habitats in the Arctic, and on glaciers and snowfields throughout the world where they thrive in high-irradiation environments [12, 13]. To overcome this high irradiance, snow algae produce secondary carotenoids resulting in blooms of red algal biomass [14], which darkens snow and ice surfaces. In Sierra Nevada snowfields, snow algae abundance was negatively correlated to surface albedo [15] and a recent study quantified the role of snow algae communities in snowmelt on an icefield in Alaska [16]. Similarly, in the Arctic, red algal blooms darken the snow/ice surface, lowering surface albedo (by as much as 13% over the melt season) [17] and increasing melt rates [18, 19].

Allochthonous material delivered to snow and ice surfaces such as forest fire-derived black carbon, Saharan or pro-glacial mineral dust, volcanic ash, and anthropogenic pollution causes increased absorption of solar radiation and locally accelerated melting. These effects can be far reaching—a darkening of the Greenland ice sheet has been observed coincident with increased melt [20]. While the effects of inorganic material on albedo have been quantified, climate models have not traditionally accounted for melting caused by snow algae [17]. These efforts are complicated by the difficulty in separating abiotic albedo from biologically induced darkening, or bio-albedo, as well as a paucity of data on snow algae distribution and density. However, a recently developed spectral model for bio-albedo which includes snow physical properties and meteorological data indicates that algal blooms can influence snowpack albedo and melt rate [19]. The model indicated that algae biomass has a greater effect than pigment concentration, suggesting a positive correlation between supraglacial algal blooms and accelerated melt. Consistent with this observation, a role for snow algae in lowering albedo through bio-geophysical feedback was also quantified on an icefield in Alaska where snow algae abundance increased with addition of either water or nutrients (a nitrogen–phosphorous–potassium fertilizer)[16].

Understanding both geologic glacial/interglacial oscillations and predicting future climate requires integrating climate models, carbon cycling, and planetary albedo. Algal clades, including green algae, evolved prior to Neoproterozoic glaciations [21]. Thus, the cosmopolitan nature of snow algae and their widespread distribution on snowpacks worldwide [22] facilitates their inclusion in these models across space and time. Snow algae is now recognized as a key component driving melting, yet the role of increasing CO_2_ on snow algae primary productivity (a proxy for growth), and thus albedo, remains unconstrained. Here we examined community composition and primary productivity (carbon fixation rates) of snow algae communities on supraglacial snowfields on glaciers on stratovolcanoes in the Pacific Northwest (USA). We targeted Gotchen Glacier on Mt. Adams, Eliot Glacier on Mt. Hood, and Collier Glacier on North Sister (Fig. 1; Table S1) where our previous data suggested photoautotrophic snow algae could be inorganic carbon-limited [23].

**Fig. 1:**
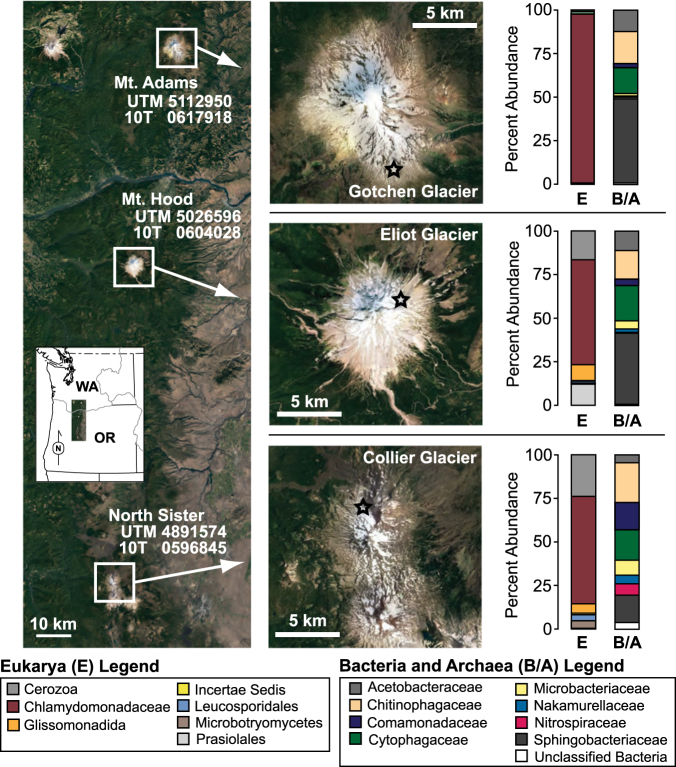
Map of sampling site locations and composition of small subunit 16S and 18S rRNA gene sequences. OTUs for each library were binned at the Family level. Only OTUs which were present in 50% or more of the samples are presented

## Stratovolcano supraglacial microbial community composition

Snow algae assemblages were comprised predominantly of eukaryotic 18 S rRNA gene sequences affiliated with *Chlamydomonas* spp. and *Chloromonas* spp. within the *Chlorophyta* (green algae)(Fig. 1). OTUs affiliated with strains of *Chlamydomonas nivalis* were abundant in supraglacial snow from Gotchen and Eliot Glaciers whereas OTUs affiliated with a *Chloromonas* spp. were the most abundant in the Collier Glacier snow sample. The sequences recovered are similar to those recovered from the Arctic, further indicating snow algae are cosmopolitan [17]. Bacterial OTUs most closely related to *Chitinophagaceae*, *Cytophagaceae*, and *Sphingobacteriaceae* were abundant in snow algae samples from the three glaciers (Fig. 1). The recovery of these bacteria is consistent with previous studies of supraglacial snow [13, 17] (Hamilton and Havig, 2017) and highlights a role for these populations in degradation of complex organic carbon on the glacial surface.

## Stratovolcano snow algae primary productivity

**Fig. 2:**
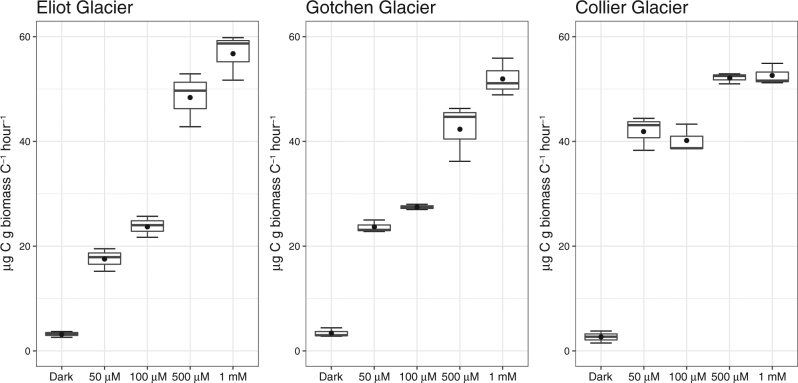
Box-Whisker plots of carbon assimilation rates by supraglacial communities. The horizontal line in each box indicates the median and closed circles represent the mean (*n* = 3 for each treatment). Dark treatments were amended with 100 µM NaH^13^CO_3_

Carbon fixation rates were examined in a series of microcosms in supraglacial snow over a range of dissolved inorganic carbon (DIC) concentrations (50 µM to 1 mM NaH^13^CO_3_) where natural DIC concentration in snow samples ranged from 10.6 to 46.3 µM (Table S1). At all sites, an increase in light-dependent carbon fixation was observed with increasing concentration of DIC concentration (Fig. 2). In microcosms amended with 50 µM NaH^13^CO_3_ (Fig. 2; Table S1), rates of carbon assimilation ranged from ~17 µg C/g C_biomass_/h in supraglacial snow algae from Eliot Glacier to ~42 µg C/g C_biomass_/h at Collier Glacier. Microcosms amended with 500 µM or 1 mM NaH^13^CO_3_ incorporated significantly more carbon than assays amended with 50 µM or 100 µM NaH^13^CO_3_ (Fig. 2; Table S1). This effect was particularly pronounced at Eliot and Gotchen Glaciers where rates increased 77–108% in the presence of elevated NaH^13^CO_3_ (50 µM vs. 1 mM). The increase in carbon assimilation rates at Collier Glacier was less pronounced—we observed an increase of ~20–25% in carbon assimilation between 50–100 µM NaH^13^CO_3_ and 500 to 1 mm µM NaH^13^CO_3_.

## Implications for future and past climate models

Snow algae lower albedo and thus can alleviate water limitation by increasing meltwater [16]. As a result, increasing snow algae abundance acts as a positive feedback mechanism to accelerate melt. Here, we present data indicating snow algae primary productivity is stimulated by the addition of CO_2_. Assuming carbon fixation is a proxy for growth, our data suggest increasing atmospheric CO_2_ concentration will increase perennial snow algae abundance, serving as an additional positive feedback mechanism between snow algae and decreased albedo. These data underscore the need to quantify algal abundance in mountain glaciers which are particularly susceptible to climate change. Furthermore, these data add to the growing calls to quantify the effects of bio-albedo and the need for integrating algal–albedo interactions and variable (increasing) CO_2_ in models aimed at interpreting Earth’s past glacial/interglacial oscillations as well as current and future climate models.

## Electronic supplementary material

Supplemental Material

## References

[1] Hoffman PF, Kaufman AJ, Halverson GP, Schrag DP (1998). A Neoproterozoic snowball earth. Science.

[2] Rasmussen B, Bekker A, Fletcher IR (2013). Correlation of Paleoproterozoic glaciations based on U–Pb zircon ages for tuff beds in the Transvaal and Huronian Supergroups. Earth Planet Sci Lett.

[3] Clark PU, Alley RB, Pollard D (1999). Northern hemisphere ice-sheet influences on global climate change. Science.

[4] Edwards A, Irvine-Fynn T, Mitchell AC, Rassner SME (2014). A germ theory for glacial systems?. Wiley Interdiscip Rev Water.

[5] Sigman DM, Boyle EA (2000). Glacial/interglacial variations in atmospheric carbon dioxide. Nature.

[6] Gough DO (1981). Solar interior structure and luminosity variations. Sol Phys.

[7] Allen PA, Etienne JL (2008). Sedimentary challenge to Snowball Earth. Nat Geosci.

[8] Caldeira K, Kasting JF (1992). Susceptibility of the early Earth to irreversible glaciation caused by carbon dioxide clouds. Nature.

[9] Waters CN, Zalasiewicz J, Summerhayes C, Barnosky AD, Poirer C, Gałuszka A (2016). The Anthropocene is functionally and stratigraphically distinct from the Holocene. Science.

[10] Wolff EW (2011). Greenhouse gases in the Earth system: a palaeoclimate perspective. Philos Trans A Math Phys Eng Sci.

[11] Anesio AM, Laybourn-Parry J (2012). Glaciers and ice sheets as a biome. Trends Ecol Evolut.

[12] Morgan-Kiss RM, Priscu JC, Pocock T, Gudynaite-Savitch L, Huner NPA (2006). Adaptation and acclimation of photosynthetic microorganisms of permanently cold environments. Microbiol Mol Biol Rev.

[13] Boetius A, Anesio AM, Deming JW, Mikucki JA, Rapp JZ (2015). Microbial ecology of the cryosphere: sea ice and glacial habitats. Nat Rev Microbiol.

[14] Remias D, Lütz-Meindl U, Lütz C (2005). Photosynthesis, pigments and ultrastructure of the alpine snow alga *Chlamydomonas nivalis*. Eur J Phycol.

[15] Thomas WH, Duval B (1995). Sierra Nevada, California, USA, snow algae: snow albedo changes, algal-bacterial interrelationships, and ultraviolet radiation effects. Arctic and Alpine Research.

[16] Ganey GQ, Loso MG, Bryant Burgess A, Dial RJ (2017). The role of microbes in snowmelt and radiative forcing on an Alaskan icefield. Nat Geosci.

[17] Lutz S, Anesio AM, Raiswell R, Edwards A, Newton RJ, Gill F (2016). The biogeography of red snow microbiomes and their role in melting arctic glaciers. Nat Commun.

[18] Musilova M, Tranter M, Bamber JL, Takeuchi N, Anesio AM (2016). Experimental evidence that microbial activity lowers the albedo of glaciers. Geochem Perspect Lett.

[19] Cook JM, Hodson AJ, Taggart AJ, Mernild SH, Tranter M (2017). A predictive model for the spectral “bioalbedo” of snow. J Geophys Res Earth Surf.

[20] Tedesco M, Doherty S, Fettweis X, Alexander P, Jeyaratnam J, Stroeve J (2016). The darkening of the Greenland ice sheet: trends, drivers, and projections (1981-2100). Cryosphere.

[21] Knoll AH (1992). The early evolution of eukaryotes: a geological perspective. Science.

[22] Hisakawa N, Quistad SD, Hester ER, Martynova D, Maughn H, Sala E (2015). Metagenomic and satellite analyses of red snow in the RussianArctic. Peer J.

[23] Hamilton TL, Havig JR (2017). Supraglacial primary productivity in glaciers on stratovolcanoes of the Pacific Northwest. Geobiology.

