# Supplementary material for: Inorganic carbon addition stimulates snow algae primary productivity
Source: ISME J. 2018 Jan 29;14(3):857–60. doi: 10.1038/s41396-018-0048-6 (PMC7031325; doi:10.1038/s41396-018-0048-6)
Supplement: Supplementary file 1 — Supplemental Material [file 41396_2018_48_MOESM1_ESM.pdf]

Inorganic carbon addition stimulates snow algae primary productivity

Trinity L. Hamilton, Jeff R. Havig

SUPPLEMENTAL MATERIAL

METHODS

Sample collection and aqueous geochemistry

Samples were collected in the July and August of 2016 from Gotchen Glacier (46.159 N -121.472 W) on Mt. Adams in Washington, USA, Eliot Glacier (45.385 N -121.671 W) on Mt. Hood in Oregon, USA, and Collier Glacier (44.170 N -121.788 W) on North Sister in Oregon, USA (See Fig. 1 in the main text for sample site locations). Sample collection and aqueous geochemistry followed methods described previously (Hamilton and Havig, 2017) except cations, anions and trace element analyses were performed by the Laboratory for Isotopes and Metals in the Environment at the Pennsylvania State University.

CO_2 photoassimilation and stable isotope signals

A microcosm-based approach was employed to assess *in situ* inorganic carbon uptake through the addition of $NaH^{13}CO_3$ as we have described previously (Hamilton and Havig, 2017). While we are aware of debate regarding validity of microcosm amendment studies for studying microbial activities in snow, particularly dry snow (i.e., Carpenter et al., 2000), we targeted areas of active melt with snow of high water content and samples were collected during the Summer months when daytime ambient temperatures were well above freezing (~12-14ºC). In our previous study (Hamilton and Havig, 2017), we observed inorganic carbon uptake following a 60-minute incubation time in ~15-mL assays amended with 100 µM $NaH^{13}CO_3$. Here we employed a similar method: The surface layer of wet snow where phototrophic populations were visibly apparent (red or orange colored snow) was collected using a sterile spatula and placed into a clean / sterile container. The snow was allowed to melt to a snow-slush slurry (~15 minutes) and was homogenized with the spatula. Approximately 20-mL of the snow-slush slurry was then transferred to clear 40-mL acid-washed screw-cap polypropylene bottles. Assays were then initiated by amending with $NaH^{13}CO_3$ (Cambridge Isotope Laboratories, Inc., Andover MA). $NaH^{13}CO_3$ stock solutions were prepared with 18.2 MΩ/cm deionized water such that 100 µL was added to each

microcosm to reach the final concentration of amendment (50 µM, 100 µM, 500 µM, and 1 mM). $NaH^{13}CO_3$ was added using a sterile syringe. Following amendment, bottles were briefly and gently shaken to distribute the label. Bottles were wrapped in foil for dark assays. Bottle control assays and assays for natural abundance received unlabeled $NaH^{13}CO_3$ prepared as described above. All assays were performed in triplicate. Bottles were pushed into areas of soft snow near the collection site such that approximately 50% of the bottle was directly exposed to sunlight and incubated for approximately 2 hours *in situ*. To stop the assays, bottles were flash-frozen on dry ice, transported on dry ice, and stored at -20°C until processing.

For processing, assays were thawed and biomass was washed with HCl (1 M) to remove any carbonate minerals and residual $NaH^{13}CO_3$, then washed with 18.2 MΩ/cm deionized water and dried (60°C for three days). Dried biomass was ground/homogenized with a clean mortar and pestle. Dried and ground samples for determination of carbon concentration and stable isotope signal were weighed and placed into tin boats and sealed. Samples were analyzed via a Costech Instruments Elemental Analyzer (EA) periphery connected to a Thermo Scientific Delta V Advantage Isotope Ratio Mass Spectrometer (IR-MS) in the Department of Geology at the University of Cincinnati. Linearity corrections were made using NIST Standard 2710. $\delta^{13}C$ values were calibrated using reference standards USGS-40 and USGS-41 and checked with a laboratory standard (glycine). All carbon stable isotope results are given in delta formation expressed as per mil (‰). Carbon stable isotopes were calculated according to the following equation:

$$\delta^{13}C= [((R_a)_{sample}/(R_a)_{standard})-1] \times 10^3, \quad (1)$$

where R_a is the $^{13}C/^{12}C$ ratio of the sample or standard, and are reported versus the Vienna Pee Dee Belemnite (VPDB) standard. Reported values of DIC uptake (carbon fixation rates) were calculated using the difference in absolute $^{13}C/^{12}C$ ratios between the labeled assays and unlabeled controls.

DNA extraction and sequence analysis

DNA was extracted in triplicate from ~200 mg of biomass using a DNeasy PowerSoil Kit (Qiagen, Carlsbad, CA) according to the manufacturer's instructions. Equal volumes of triplicate extractions were pooled and the concentration of DNA was determined using a Qubit dsDNA HS Assay kit and a Qubit 3.0 Fluorometer (Invitrogen, Burlington, ON, Canada). Negative control extractions consisted of 18.2 MΩ/

cm deionized water placed in sterile 1.5-ml microcentrifuge tubes in the field or no sample (extraction blank). No DNA was detected in the control using the Qubit dsDNA HS Assay kit and sequencing failed to generate amplicons (see below for amplicon sequencing details).

For archaeal and bacterial 16S rRNA gene sequencing, total DNA was submitted to the Center for Bioinformatics & Functional Genomics at Miami University (Oxford, OH, USA). Bacterial and archaeal 16S rRNA sequences were targeted using the primers (515f) (Caporaso et al., 2012) and 806rB (Apprill et al., 2015). For eukaryotic 18S rRNA sequencing, total DNA was submitted to the Centre for Comparative Genomics and Evolutionary Bioinformatics (CGEB) at Dalhousie University (Halifax, Nova Scotia, Canada). Eukaryotic 18S rRNA sequences were targeted using the primers (E572F) and (E1009)(Comeau et al., 2011). Amplicons were sequenced using MiSeq Illumina 2 × 300 bp chemistry. Each sample was sequenced once. Post sequence processing was performed within the Mothur (ver. 1.39.3) sequence analysis platform (Schloss et al., 2009) following the MiSeq SOP (Kozich et al., 2013). Read pairs were assembled and resulting contigs with ambiguous bases were removed. Contigs were trimmed to include only the overlapping regions and unique sequences were aligned against a SILVA-based reference alignment and classified using a Bayesian classifier within Mothur against the against the Silva (v128) reference taxonomy. Chimeras were identified and removed using UCHIME (Edgar et al., 2011). Sequences were classified Operational taxonomic units (OTUs) were assigned to all classified sequences at a sequence similarity of 97.0% for archaea and bacteria and 98% for eukarya using the average-neighbor algorithm. Rarefaction was calculated within mothur and based on rarefaction analysis, >95% of the predicted 16S and 18S rRNA gene diversity was sampled at this depth of sequencing (data not shown). Results were visualized with the Phyloseq R package (ver. 1.16.2; McMurdie and Holmes, 2013) and the ampvis R package (Albertsen et al., 2015)(R version 3.2.4). Sequence data including raw reads, quality scores and mapping data have been deposited in the NCBI Sequence Read Archive (SRA) database with the BioProject number PRJNA395733.

Table S1. Aqueous and bulk geochemistry of snow and snow algae samples and carbon assimilation by snow algae communities. All carbon isotope values given as absolute values. All carbon isotope values given vs. VPDB.

	Gotchen Glacier Mt. Adams, WA	Eliot Glacier Mt. Hood, OR	Collier Glacier North Sister, OR
GPS			
10 T	0617918	0604028	0596845
UTM	5112950	5026596	4891574
Error (m)	3	3	3
Elevation (m)	2183	2128	2284
Water geochemistry			
pH	4.7	5.3	4.5
Conductivity (µS/cm)	15.71	2.56	1.95
DIC Concentration (µM)	23.48	46.29	10.64
DIC $\delta^{13}C_{VPDB}$ (‰)	-14.57	-27.21	*BDL*
Bulk snow algae biomass			
Total C	5.94%	0.35%	0.48%
$\delta^{13}C_{VPDB}$	-24.19 ‰	-26.95 ‰	-24.02 ‰
Microcosms[a,b]	µg C uptake/g $C_{biomass}$ /hr		
Dark + 100 µM	3.39 ± 0.83	3.50 ± 0.32	2.66 ± 1.15
50 µM	23.63 ± 1.20	17.52 ± 2.17	41.91 ± 3.23
100 µM	29.60 ± 1.76	23.81 ± 1.98	40.19 ± 2.66
500 µM	42.40 ± 5.46	48.49 ± 5.14	52.14 ± 0.96
1000 µM	51.98 ± 3.58	56.69 ± 4.40	52.56 ± 2.01

[a] Dark = aluminum foil wrapped
[b] Final concentration of added ^{13}C bicarbonate.
BDL = below detection limits for the analytical technique.

Table S2. Accession numbers for the 16S and 18S rRNA amplicon libraries included in the present study.

Accession	BioProject	Site	Library name (16S rRNA)	Library name (18S rRNA)
SAMN07414146	PRJNA395733	Gotchen Glacier	728C_16S	728C_18S
SAMN07414147	PRJNA395733	Eliot Glacier	730A_16S	730A_18S
SAMN07414148	PRJNA395733	Collier Glacier	806G_16S	806G_18S

REFERENCES

Albertsen M, Karst SM, Ziegler AS, Kirkegaard RH, Nielsen PH. (2015). Back to basics–the influence of DNA extraction and primer choice on phylogenetic analysis of activated sludge communities. *PLoS ONE* **10**:e0132783. (doi:10.1371/journal.pone.0132783)

Apprill A, McNally S, Parsons R, Weber L. (2015). Minor revision to V4 region of SSU rRNA 806R gene primer greatly increases detection of SAR11 bacterioplankton. *Aquat Microb Ecol* **75**:129-137. (doi: 10.3354/ame01753)

Caporaso JG, Lauber CL, Walters WA, Berg-Lyons D, Huntley J, Fierer N, et al. (2012) Ultra-high-throughput microbial community analysis on the Illumina HiSeq and MiSeq platforms. *ISME J* **6**:1621–1624. (doi:10.1038/ismej.2012.8)

Carpenter EJ, Lin S, Capone DG. (2000) Bacterial Activity in South Pole Snow. *Appl Environ Microbiol* **66**:4514-4517. (doi:10.1128/AEM.66.10.4514-4517.2000)

Comeau AM, Li WKW, Tremblay J-é, Carmack EC, Lovejoy C. (2011). Arctic Ocean microbial community structure before and after the 2007 record sea ice minimum. *PLoS ONE* **11**:e27492. (doi: 10.1371/journal.pone.0027492)

Edgar RC, Haas BJ, Clemente JC, Quince C, Knight R. (2011). UCHIME improves sensitivity and speed of chimera detection. *Bioinformatics* **27**:2194–2200. (doi:10.1093/bioinformatics/btr381)

Hamilton TL, Havig JR. (2017). Supraglacial primary productivity in glaciers on stratovolcanoes of the Pacific Northwest. *Geobiology* **15**:280–295. (doi:10.1111/gbi.12219)

Kozich JJ, Westcott SL, Baxter NT, Highlander SK, Schloss PD. (2013). Development of a dual-index sequencing strategy and curation pipeline for analyzing amplicon sequence data on the MiSeq Illumina sequencing platform. *Appl Environ Microbiol* **79**:5112–5120. (doi:10.1128/AEM.01043-13)

McMurdie PJ, Holmes S. (2013). phyloseq: an R package for reproducible interactive analysis and graphics of microbiome census data. *PLoS One* **8**: e61217. (doi:10.1371/journal.pone.0061217)

Schloss PD, Westcott SL, Ryabin T, Hall JR, Hartmann M, Hollister EB, et al. (2009). Introducing mothur: open-source, platform-independent, community-supported software for describing and comparing microbial communities. *Appl Environ Microbiol* **75**:7537–7541. (doi:10.1128/AEM.01541-09)
